# Drs. Sayre and Jacobi on Homœopathy and Homœopathists

**Published:** 1883-03

**Authors:** 


### DRS. SAYRE AND JACOBI ON HOMŒOPATHY AND HOMŒOPATHISTS.

While discussing the "new code," the allopathists denounce Homœopathy; one declares there are not more than two or three homœopathists in New York who believe in Hahnemann. The other affirms that homœopathists do not now believe their law to be anything but a convenient rule—occasionally applicable!

These gentlemen must surely read the reports of the American Institute! They seem to be as well informed about Homœopathy as the late presidents of that noble body, one of *whom is here quoted in evidence against Homœopathy*—which is about the only thing he is good for!

"What is your particular objection to the new code?" asked the reporter.

Dr. Sayre: "Why, the clause permitting consultations with all legally qualified physicians amounts to nothing more nor less than a compromise with the so-called homœopathists, and such a course excommunicates our association from the rest of the medical world. Clairvoyants, mesmerists, hydropathists, electricians, and all the other myths of medicines may call themselves legally qualified physicians. Most of the regular physicians who have effected this concession to the homœopathists did not do so out of any respect for Homœopathy, as they themselves will tell you. They despise the doctrines of Homœopathy as thoroughly as I do."

"Then what is their purpose, Dr. Sayre?"

"They claim that they will effectually stamp out the theories of Homœopathy as soon as they are allowed to get at its advocates. I admit that there was a time when the denunciation of Hahnemann's system of therapeutics by the body of regular physicians was an indirect assistance to the homœopathists. The latter knew that their methods at that time were superior to the overdosing and heroic methods of the regular physicians, and they were only too glad to be challenged to the proof. Their mild treatment was certainly an improvement upon the very severe treatment of many diseases as it then existed. But times have changed. Regular physicians are giving less medicine and homœopathists are giving more. I can see no advantage in making any concessions to them now. Some other physicians—specialists—look at the matter in another light. These men have acted like a lot of quacks themselves. They have divided up the profession. One man must attend to your eye, another to your ear, another to your nose, another to your upper lip, and another to your big toe. Now, I admit the value of a particular line of study and practice, such as I have myself, but I am not a specialist—I am a doctor. A man has no right to call himself a physician unless he is familiar with the entire body and the diseases to which it is subject. A corn on your foot more or less affects all the other parts of the body, and until a man has become familiar with the effects of all the diseases of the body he certainly has no right to dub himself a specialist. Now, then, Homœopathy in the abstract has a considerable *clientèle* among the wealthy inhabitants of New York. By accepting consultations with homœopathic physicians these specialists come in for a share of the fees which wealthy patients are ready to pay. Some of my fellow practitioners have thought me a fool because I did not see that in my own particular line of practice I could reap great profits through the system of free consultations. Maybe I could, but I am not prepared to throw away my honest convictions for an increased income, and in my opinion Homœopathy is the biggest humbug of the age.

"Homœopathy," Dr. Sayre went on, "is not Homœopathy. I don't believe there are more than two or three physicians in the city who believe in the dogmas of Hahnemann, as laid down in his *Organon.* I ask any so-called homœopathist to come out boldly and acknowledge his faith in them, and I pause for a reply. I don't believe there is one who will confess to such a faith. I should like to put another question—Is there one homœopathic physician in New York to-day who really believes in the increased power of the thirtieth and sixtieth solution? I don't suppose you know what that means, do you? No more do I; but it is intended to mean that the more you dilute a thing the more powerful it becomes. No man who has preserved his reason can accept such a theory, and no man can profess such a theory while really disbelieving it without becoming a quack. *The doctors who reject the theories of Hahnemann and still call themselves homœopathists are imposing upon the public.* I have studied all there was to study of Homœopathy, and I know just what I am talking about. I could no more hold a consultation with a homœopathic physician than I could with a—with a—well, I could not do it, and that is all,* and I want every regular doctor in the County Medical Society to go on record on this question."

Dr. Abraham Jacobi: "I used to favor a code of ethics because I believed that young men in the profession would be guided by a written law. I find that I was mistaken, and I am now in favor of no code at all. A code of ethics never made a gentleman and it never will. As between the old code and the new code I favor the new one decidedly. I am not a specialist; I am not an allopathist; I am a regular physician. I object to the name allopathist. It was a term invented by Hahnemann to denote the opponents of his alleged discovery of *similia similibus curantur.* I am as heartily opposed to the doctrines of Homœopathy as any one, but I do not see that there is much left to oppose. Homœopathy does not amount to enough, in my opinion, to deserve opposition. Let any one read Dr. Dowling's article on this subject in the *North American Review* some months ago and he will be convinced of what I say, that there is nothing left of Homœopathy but the doctrine of 'like cures like.' Even that, as the homœopathists will admit, is no longer laid down as a general law, but as a rule which does not hold good in all cases. No school of medicine can be founded on a law which is not general. I never consulted with a homœopathist in my life until lately, when the doctor who was said to be a homœopathist told me that he was not such in any strict sense; that he was only a doctor; and I discovered later on that he was not much of a doctor either. Homœopathy is something which exists only in an abstract way in a portion of the public mind. You will hear some old ladies discussing it on the balcony of a summer hotel, and they will get into a temper in discussing it just as they would over a discussion of religion. It is a mere matter of faith with them. Homœopathists have thrived upon persecution, though I think there has been less persecution than they claim. I say, take down the fence that has shut them off from the body of regular physicians. Medicine should not recognize any particular sect; and if these disciples of Hahnemann, who are

---

* Affidavits against Dr. Sayre. One of the delegates who represented New York County in the State Convention, and who, with the twenty-three other delegates, favors the new code, declared to the reporter that affidavits had been prepared to prove at least two cases in which Dr. Sayre had consulted with homœopathic physicians!

only so in name, who hang on to the appellation simply as a trade-mark, want to come into the fold, why should we hinder them?"—*N. Y. Herald*, January 28th, 1883.

